# Intercontinental transmission of West Nile virus by migrating white storks.

**DOI:** 10.3201/eid0707.017719

**Published:** 2001

**Authors:** M Malkinson, Y Weisman, S Pokamonski, R King, V Deubel

**Affiliations:** Kimron Veterinary Institute, Beit Dugan, Israel. martinm@moag.gov.il


Table 2. Plague in the Americas: Reservoirs and Vectors

| Country | Reservoirs | Vectors |
|---|---|---|
| Bolivia | *Akodon* sp | *Xenopsylla cheopis* |
| | *Rattus rattus* | *Pulex irritans* |
| Brazil | *Akodon* sp | *X. cheopis* |
| | *Oryzomys* sp | |
| | *Callomys* sp | |
| | *Bolomy* sp | |
| | *Monodelphis deomestica* | |
| Ecuador | *R rattus* | *P. irritans* |
| | *R. norvegicus* | |
| | *R. alexandrinus* | |
| | *Akodon mollis* | |
| | *Oryzomys* sp | |
| | *Phyllotis* sp | |
| | *Scirurus stramineus* | |
| United States | Marmot (*Cynomys* sp) | *Orchopeas sexdentatus* |
| | Rabbits | *Oropsylla montana* |
| | Rats (*Dipodomys* sp) | *Haplosyllus* sp |
| | Mice (*Peromyscus* sp) | *Diamanus* sp |
| | Terrestrial squirrel (*Citellus* sp) | *Thrasis* sp |
| Peru | *Akodon* sp | *X cheopis* |
| | *Oryzomys* sp | *Polygenes* sp |
| | *Sigmodon* sp | *Tiamastus* sp |
| | *Phyllotis* sp | *P. irritans* |
| | *R. rattus* | |
| | *Cavia porcellus* | |

where plague is prevalent because dogs are susceptible to *Y. pestis* infection. Although they rarely develop the disease, they can maintain detectable titers of antibodies for extended periods. Trapping rodents can also be used for surveillance to detect *Y. pestis* infection by microbiologic or serologic testing and for identifying the flea vectors.

Identifying and treating infected persons are priorities in plague-endemic areas. Streptomycin is the most effective antibiotic for treating plague. Tetracyclines are preferred for prophylactic use. Vaccination is not possible because no effective vaccines currently exist.

Education is appropriate in the areas where infection is known and where people are at risk. Messages can be delivered that take into account the local, cultural, and ethnic characteristics of the communities.

Flea surveillance and control with proper insecticides could be carried out by a local community. Periodic application of insecticides inside and outside homes is important in reducing the flea population in infected areas. Other prevention measures could be implemented on the basis of local risk assessments; for example, in Peru when improper storage of grains attracted rodents inside the houses, small silos were designed to store the goods.

We recognize that plague is still in the Americas and human population is rapidly growing. New lands are being used for new settlements, and new crops are being grown for food production. Many species of rodents can serve as reservoirs, not only for *Y. pestis* infection but also for other emerging infections, and at any moment a new outbreak might appear. Local, cross-cutting, and interdisciplinary approaches are encouraged to implement adequate surveillance of rodentborne diseases.

### Intercontinental Transmission of West Nile Virus by Migrating White Storks

**Mertyn Malkinson,* Caroline Banet,* Yoram Weisman,* Shimon Pokamonski,† Roni King,‡ and Vincent Deubel§**

*Kimron Veterinary Institute, Beit Dagan, Israel; †Israeli Veterinary Services, Beit Dagan, Israel; ‡Nature Reserves Authority, Israel; and §Pasteur Institute, Paris, France

In September and October 1998, West Nile (WN) virus was isolated from a flock of 1,200 migrating white storks (*Ciconia ciconia*) that had landed in Eilat, a town in southern Israel. Inclement weather conditions of strong, hot westerly winds had forced them to fly under considerable physical stress to reach Eilat. The storks were fledgelings, less than 1 year old, that had hatched in Europe. Analysis of blood samples taken from several birds within days of their arrival showed the presence of WN virus–neutralizing antibodies. Sequence analysis of the envelope glycoprotein gene of the stork isolate showed almost complete identity with a sample isolated from a dead goose in Israel in 1998.

Because this Eilat flock was migrating southward for the first time and had not previously flown over Israel, we assume that it became infected with WN virus in Europe. The presence of virus-neutralizing antibodies in stork serum samples collected from German flocks provided additional evidence that the birds contracted WN virus in Europe. These findings indicate that the recent epizootic of WN virus in Israeli geese had its origin in Europe, where the virus had been circulating in epidemic proportions since 1996. Epidemiologic studies of eastern European epidemics indicate that WN virus may now be endemic in southern Europe.

Address for correspondence: Mertyn Malkinson, Kimron Veterinary Institute, Beit Dagan, P.O. Box 12, Israel 50250; fax: 972-3-9681739; e-mail: martinm@moag.gov.il

